# Lenvatinib-Induced Glomerular Microangiopathy in a Patient With Poorly Differentiated Thyroid Carcinoma: A Long-Term Renal Function Change With Biopsy Findings

**DOI:** 10.7759/cureus.91970

**Published:** 2025-09-10

**Authors:** Takuya Ishimura, Hiroyuki Suzuki, Tomomi Endo, Takeshi Matsubara, Toshiki Maetani, Eri Muso, Tatsuo Tsukamoto

**Affiliations:** 1 Department of Nephrology and Dialysis, Medical Research Institute Kitano Hospital, PIIF Tazuke-Kofukai, Osaka, JPN; 2 Division of Nephrology and Dialysis, Ozawa Hospital, Odawara, JPN; 3 Department of Otolaryngology, Head and Neck Surgery, Medical Research Institute Kitano Hospital, PIIF Tazuke-Kofukai, Osaka, JPN; 4 Department of Food and Nutrition, Faculty of Contemporary Home Economics, Kyoto Kacho University, Kyoto, JPN

**Keywords:** glomerular microangiopathy, lenvatinib, nephrotic syndrom, onconephrology, thyroid tumor

## Abstract

Lenvatinib, a multi-targeted tyrosine kinase inhibitor (TKI), is widely used in the treatment of radioactive iodine-refractory thyroid carcinoma, including poorly differentiated subtypes. While its clinical efficacy is well documented, significant adverse events, such as proteinuria and renal dysfunction, may confine the treatment. This report describes a case of a 77-year-old man with poorly differentiated thyroid carcinoma who developed nephrotic-range proteinuria and progressive renal impairment shortly after initiating lenvatinib. Renal biopsy revealed histological features consistent with glomerular microangiopathy (GMA), including mesangiolysis, endothelial swelling, and double-contour formation of glomerular capillaries. Following the temporary discontinuation of lenvatinib, proteinuria and renal function gradually recovered. However, due to the drug’s significant antitumor effects, lenvatinib was restarted at a reduced dose and titrated cautiously. Despite persistent proteinuria and a gradual decline in renal function, treatment was continued for 43 months. Lenvatinib was ultimately discontinued when serum creatinine reached 4.53 mg/dL. After cessation, renal function stabilized without the need for renal replacement therapy, but the patient died of progressive metastatic disease. This case highlights the risk of lenvatinib-induced GMA and the importance of renal biopsy in confirming the diagnosis. It also demonstrates the complex clinical decision-making required to balance oncologic efficacy with renal safety. Careful dose adjustment and close monitoring of renal parameters are essential in patients who develop nephrotoxicity during TKI therapy. This report adds to the growing evidence on lenvatinib-induced nephropathy and underscores the need for individualized treatment strategies in patients with advanced thyroid carcinoma.

## Introduction

Malignant tumors remain a leading cause of mortality worldwide, with an estimated 20 million new cancer cases diagnosed in 2022. Among these, thyroid cancer ranks seventh, accounting for 4.1% of all malignancies [1]. Although the overall prognosis for thyroid cancer is generally favorable, contributing to only 0.8% of cancer-related deaths, poorly differentiated thyroid carcinoma (PDTC) demonstrates intermediate biological behavior between well-differentiated and anaplastic carcinomas and significantly worse outcomes because of its radioactive iodine (RAI)-refractory characteristics [2]. Tyrosine kinase inhibitors (TKIs) have emerged as effective therapeutic options for malignancies resistant to conventional treatment. Lenvatinib, a multi-targeted TKI, is approved for the management of unresectable thyroid carcinoma, hepatocellular carcinoma, renal cell carcinoma, and recurrent endometrial cancer. Its introduction has significantly improved treatment outcomes in several difficult-to-treat cancers [3]. However, its clinical use is sometimes complicated by adverse events, including hypertension, diarrhea, fatigue, and especially proteinuria [3]. Lenvatinib-induced proteinuria is a major adverse effect, reported in 31% of all grades and 10% of grade 3 or higher [4]. While severe proteinuria can necessitate dose reduction or discontinuation of therapy, long-term renal outcomes with histological confirmation in PDTC patients are underreported. We here demonstrate a case of massive proteinuria in a patient with PDTC undergoing lenvatinib therapy. Lenvatinib is known to induce a specific type of microvascular dysfunction (glomerular microangiopathy (GMA)) by inhibiting vascular endothelial growth factor (VEGF), leading to proteinuria and renal impairment [5]. After making a diagnosis of lenvatinib-induced GMA, we could use lenvatinib at a reduced dose for our patient, leading to prolonging the relatively long survival. This case report assists in adjusting the dosage of lenvatinib in cases presenting with proteinuria, thereby contributing to improved patient prognosis.

## Case presentation

A 77-year-old male patient initially presented to our institution six years prior with dizziness. During the initial evaluation, carotid artery echography incidentally identified a 39 mm mass in the right thyroid lobe. Fine needle aspiration (FNA) was suggestive of follicular thyroid carcinoma, and the patient was placed under careful monitoring. Approximately eight months prior to hospitalization, the tumor exhibited rapid growth with mediastinal extension, bronchial compression, left vocal cord paralysis with hoarseness, and multiple pulmonary nodules suspicious for metastasis. The patient underwent total thyroidectomy and superior mediastinal lymphadenectomy four months before admission. Histopathological analysis revealed follicular carcinoma with foci of poorly differentiated carcinoma. The tumor was staged as cT4aN1bM1, stage IVc per the Union for International Cancer Control (UICC) sixth edition. Lenvatinib was initiated at 24 mg/day one month before admission, and the treatment led to a marked reduction in pulmonary metastases. However, two weeks after treatment initiation, the patient suddenly developed proteinuria, hypertension (systolic blood pressure increased from 140 mmHg to 170 mmHg), and lower limb edema. Over the subsequent 10 days, generalized edema worsened, accompanied by loss of appetite and fatigue. Seven days prior to admission (four weeks after initiation of lenvatinib), laboratory investigations revealed a urinary protein/creatinine ratio of 8596.7 mg/gCr with 3+ hematuria (dysmorphic RBC with RBC casts) and elevation of creatinine from 1.15 mg/dL to 1.75 mg/dL, prompting discontinuation of lenvatinib and initiation of oral furosemide at 40 mg. Subsequently, this resulted in increased urine output and ameliorated systemic edema with decreased body weight. However, due to the therapeutic benefit of lenvatinib, a renal biopsy was performed to investigate the etiology of the massive proteinuria and guide further management.

The patient’s medical history included hypertension managed with irbesartan (100 mg/day) and amlodipine (5 mg/day) for several years. Due to the total thyroidectomy, he takes levothyroxine (200 μg daily) for hypothyroidism and alfacalcidol (1 μg daily) for hypoparathyroidism. His baseline body weight was 74 kg, which increased to 82.5 kg following lenvatinib initiation. After discontinuation of lenvatinib and start of furosemide administration, his weight improved to 77.0 kg. On admission, his blood pressure was 139/82 mmHg, and his body temperature was within normal limits. He had neither prior urinary abnormalities nor a family history of renal diseases. Physical examination revealed bilateral lower limb edema and postoperative changes in the cervical and anterior chest regions, with no other significant findings. Laboratory investigations revealed elevated serum creatinine (1.40 mg/dL) compared to baseline, mild hypoalbuminemia (3.6 g/dL), and normocytic anemia (hemoglobin 11.8 g/dL) without leukopenia or thrombocytopenia (Table 1).

**Table 1:** Laboratory examination results on renal biopsy

Variables	At renal biopsy	Normal range
Total protein, g/dL	5.8	6.6-8.1
Albumin, g/dL	3.6	4.1-5.1
Total cholesterol, mg/dL	229	142-248
Urea nitrogen, mg/dL	17.3	8.0-20.0
Creatinine, mg/dL	1.40	0.65-1.07
C reactive protein, mg/dL	0.25	0-0.14
White blood cell, /µL	5000	3300-8600
Hemoglobin, g/dL	11.8	13.7-16.8
Platelet, /µL	178000	158000-348000
Immunoglobulin G (IgG), mg/dL	936	861-1747
Immunoglobulin A (IgA), mg/dL	158	93-393
Immunoglobulin M (IgM), mg/dL	63	33-183
Complement 3 (C3), mg/dL	114	73-138
Complement 4 (C4), mg/dL	41	11-31
Urinary protein, g/day	3.58	0.03-0.12
Urinary occult blood	±	-
Urinary pH	6.5	4.5-7.5

**Figure 1:**
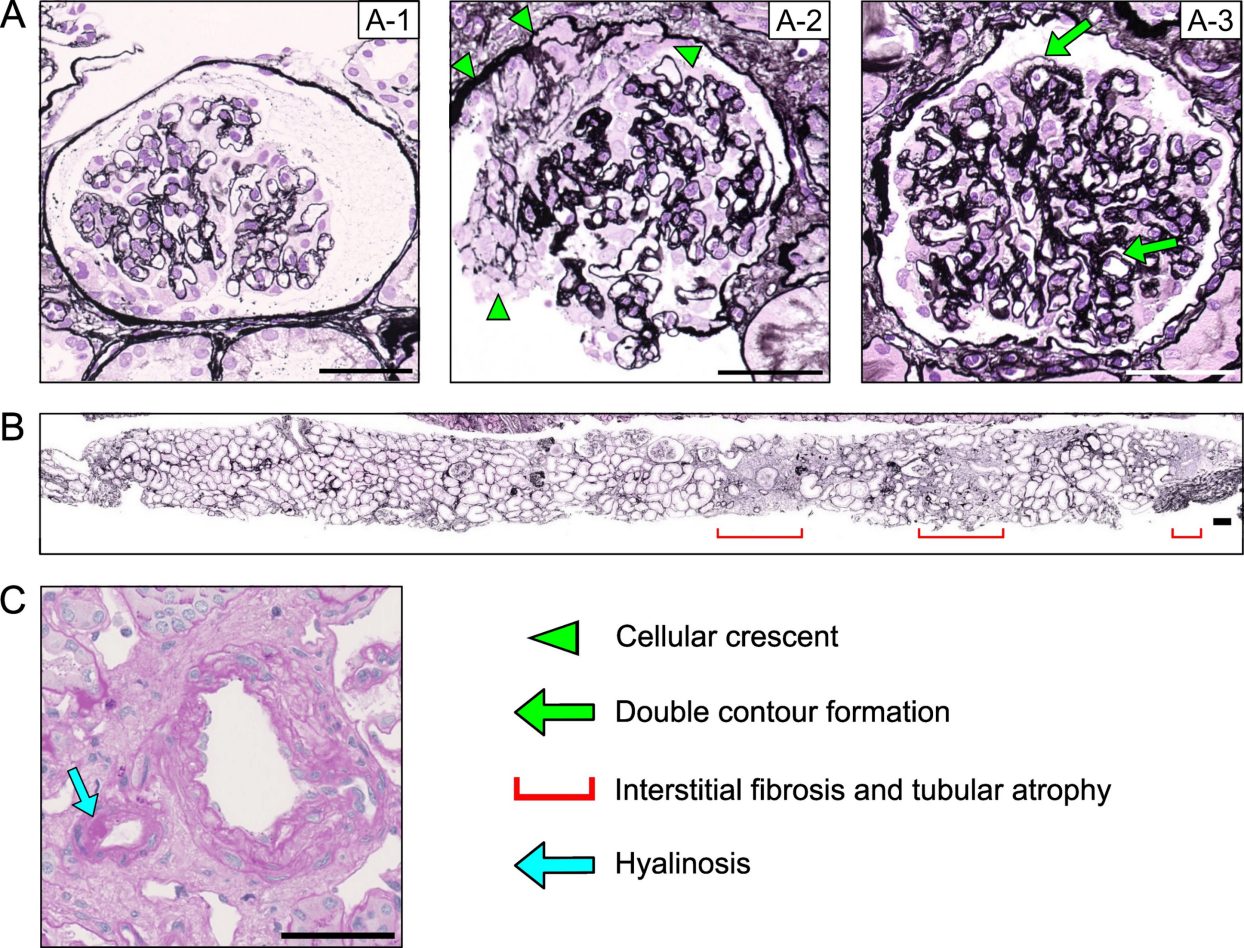
The light microscopy images of renal biopsy specimen. (A) The representative images of glomeruli. Several glomeruli exhibited mesangiolysis and widening of subendothelial space (Panel A-1), and cellular crescent with disruption of glomerular basement membrane (Panel A-2, arrowhead). Most glomeruli showed double contour formation with mild mesangial expansion (Panel A-3, arrow). (B) The representative images of interstitial space. Red line shows tubular atrophy and interstitial cellular infiltration with interstitial fibrosis. (C) The vascular images of renal biopsy. The arrow shows hyalinosis. No obvious thrombus was observed. The scale bar shows 50 μm in Figures 1A-1C, and 100 μm in Figure 1B. Periodic acid-methenamine (PAM) silver staining was used in Figures 1A-1B, and periodic acid Schiff (PAS) staining was used in Figure 1C.

The renal biopsy was performed without any complications, and the specimen contained 22 glomeruli with six global sclerotic glomeruli. Cellular crescents were identified in three glomeruli, and one glomerulus had a fibrous crescent. Several glomeruli exhibited acute GMA findings, including mesangiolysis and widening of the subendothelial space (Figure 1, Panel A-1), cellular crescent with disruption of the glomerular basement membrane (Figure 1, Panel A-2). However, most glomeruli showed only double contour formation with mild mesangial expansion (Figure 1, Panel A-3). In the interstitial area, tubular atrophy and inflammatory cell infiltration with interstitial fibrosis were focally observed (Figure 1B). Mild arteriosclerotic changes in arterioles and small arteries were observed, without apparent thrombus (Figure 1C).

**Figure 2:**
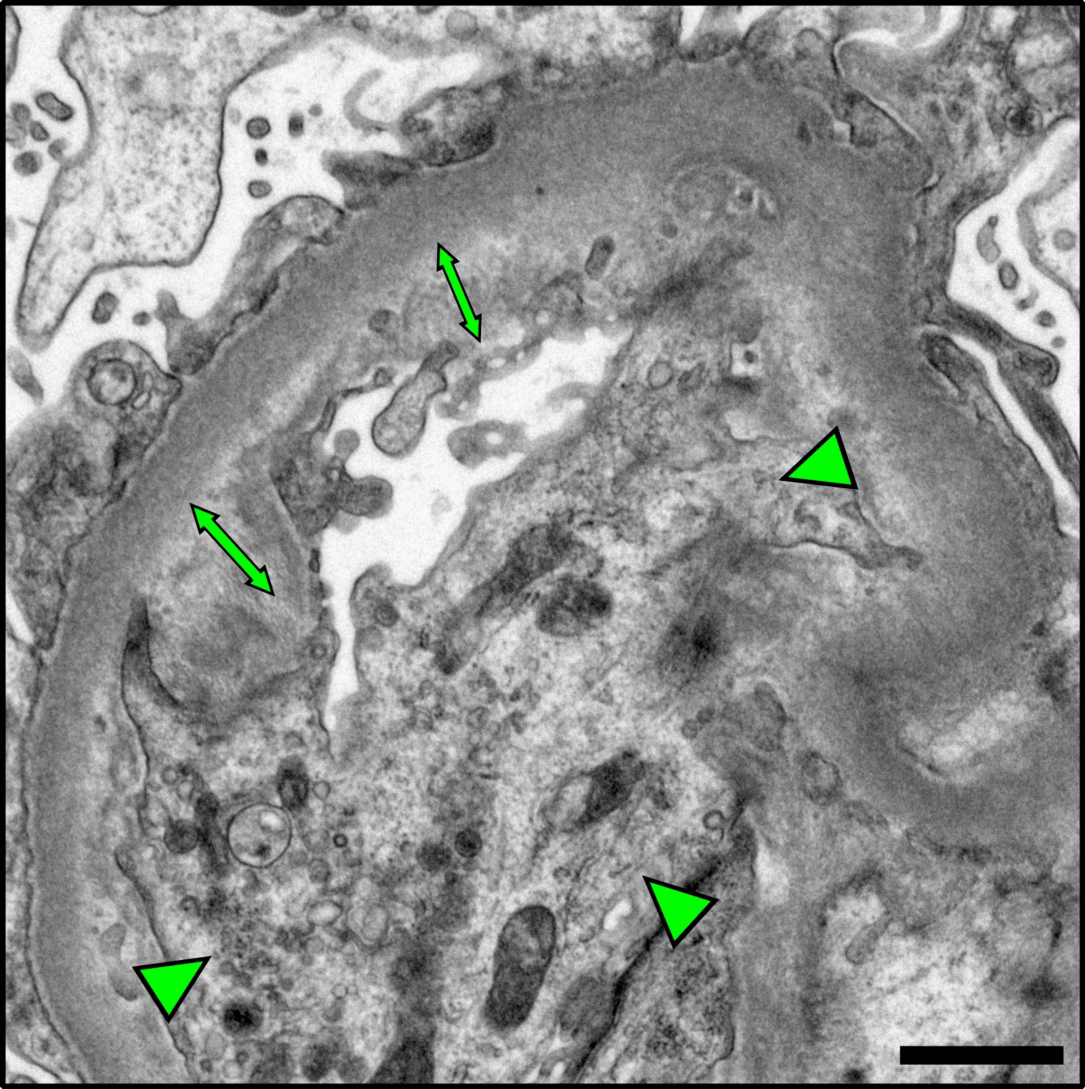
The electron microscopy images of renal biopsy specimen. The glomerular image of electron microscopy. Marked widening of subendothelial space (double-headed arrow) and endothelial cell swelling (arrowheads) were noted. The scale bar shows 1 μm.

These features were consistent with GMA, likely secondary to lenvatinib. Immunofluorescence study demonstrated no immunological findings (data not shown). Electron microscopy revealed foot process effacement, swelling of glomerular endothelial cells, and widening of the subendothelial space (Figure 2).

**Figure 3:**
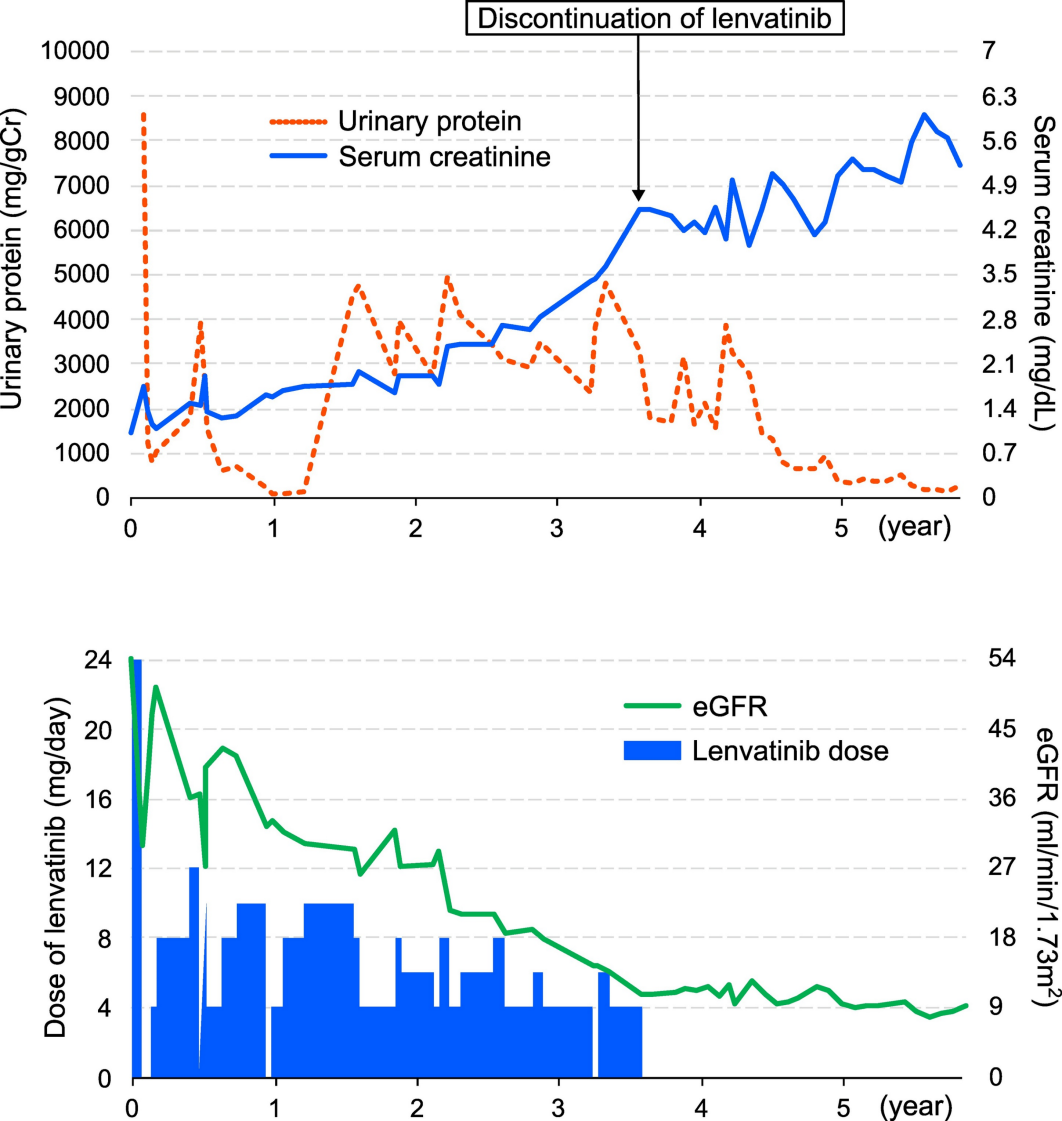
The time course of urinary protein, creatinine, and estimated glomerular filtration rate (eGFR) with lenvatinib dose. The therapeutic course. The graph shows the urinary protein, creatinine, eGFR, and dose of lenvatinib.

Three weeks after discontinuation of lenvatinib, proteinuria improved to 822.4 mg/gCr, and serum creatinine levels improved to 1.16 mg/dL. Given its marked efficacy in tumor control, lenvatinib was reintroduced at 4 mg/day. The drug dose was increased biweekly, reaching 12 mg over five weeks. However, the patient again developed edema and dyspnea, necessitating treatment interruption. Although lenvatinib treatment induced proteinuria and impaired renal function, continued administration at a reduced dose was chosen in order to prioritize its therapeutic efficacy. Finally, his creatinine was elevated to 4.53 mg/dL, and lenvatinib treatment was terminated 43 months after initiation. The time course of urinary protein and creatinine with lenvatinib dose is shown in Figure 3.

He did not wish to continue lenvatinib therapy under renal replacement therapy. Cessation of therapy stabilized renal function with 4.5 mg/dL of creatinine and could avert dialysis initiation, but metastatic disease progressed, and the patient died from cancer-related complications. The clinical summary of this case is presented in Table 2.

**Table 2:** The short clinical summary of this case

Clinical summary of this case	
The bottom of serum creatinine	1.02 mg/dL
The peak of serum creatinine	6.00 mg/dL
Time to proteinuria	15 days
The duration of lenvatinib treatment	43 months
The survival duration from initiation of lenvatinib	72 months
The survival duration from cessation of lenvatinib	29 months

## Discussion

We report a case of severe proteinuria and progressive renal dysfunction induced by lenvatinib in a patient with PDTC. Renal biopsy revealed GMA, suggesting that endothelial damage from TKI treatment was the primary pathological mechanism. Highly differentiated thyroid epithelial cell-derived tumors, including papillary and follicular carcinomas, typically exhibit favorable prognoses and respond to RAI therapy. In contrast, RAI-refractory thyroid tumor frequently shows a severe prognosis. PDTC represents a histological and clinical intermediate between well-differentiated and anaplastic thyroid carcinomas. Although PDTC can retain some iodine uptake capacity, it generally fails to respond to RAI therapy, and this lack of therapeutic response correlates with limited survival benefits [6]. Thus, the primary treatment for PDTC is a total thyroidectomy with lymph node dissection for a resectable tumor [7]. In this decade, molecular targeted therapies, including lenvatinib and sorafenib, have been approved for the treatment of RAI-refractory or unresectable thyroid carcinomas [4]. Lenvatinib is a novel multi-TKI, which inhibits FGFR-1, -2, -3, -4, VEGFR-1, -2, -3, PDGFRβ, RET, and c-KIT [8]. In the SELECT trial, a placebo-controlled and randomized study, lenvatinib significantly prolonged progression-free survival in RAI-refractory differentiated thyroid cancer (18.3 months vs. 3.6 months with placebo) [9]. Despite its therapeutic efficacy, lenvatinib is associated with a high incidence of adverse effects, including hypertension, diarrhea, anorexia, and proteinuria. Among TKIs, lenvatinib has been reported to cause proteinuria more frequently than agents such as sorafenib or vandetanib, particularly in Asian populations [10]. In fact, several studies suggest a higher incidence of TKI-associated proteinuria in Asians, necessitating close monitoring of renal parameters during treatment [11]. Proteinuria is a well-known adverse event of VEGF pathway inhibition, and nephrotic-range proteinuria (>3.5 g/gCr) occurs in approximately 1-5.3% of patients treated with lenvatinib [12]. Previous reports have documented that lenvatinib-induced glomerular injuries showed pathological changes, such as focal segmental glomerulosclerosis (FSGS) [13,14] and GMA [15]. Mechanistically, VEGF and PDGF inhibition disrupts glomerular endothelial integrity, leading to microvascular injury. While collapsing FSGS has been proposed to result from chronic ischemia secondary to GMA [16], lenvatinib-induced GMA and FSGS would be a series of pathological conditions. In our present study, the renal biopsy revealed typical features of GMA, including mesangiolysis, double contour formation, and endothelial cell swelling. These findings support the hypothesis of endothelial injury resulting from inhibition of angiogenic pathways by lenvatinib. Despite the chronic findings, including double contour formation or interstitial fibrosis, our patient was able to continue long-term lenvatinib treatment without renal replacement therapy, demonstrating the efficacy of lenvatinib dose adjustment. In this case, no obvious thrombus was observed, unlike common TMA. Since some previous reports mentioned that bevacizumab-associated GMA showed histopathological pseudothrombotic pattern [17,18], our case might show hyaline occlusive microangiopathy. However, discontinuation of lenvatinib prior to renal biopsy might have resulted in resolution of the occlusion. Although proteinuria improved rapidly after lenvatinib discontinuation, renal function recovery was incomplete following long-term exposure. While a previous report demonstrated that no significant deterioration of kidney function in patients with proteinuria treated by axitinib [19], several reports showed that prolonged lenvatinib treatment in thyroid cancer patients resulted in a significant decline in estimated glomerular filtration rate (eGFR), with the degree of proteinuria correlating with renal function deterioration [20,21]. Our case also showed a remarkable decrease at the first time of cessation, discontinuation of lenvatinib no longer improved urinary protein excretion or renal function after long-term administration. While previous reports have shown that switching to sorafenib was effective in reducing urinary protein [22], our case continued administration at a reduced dose in order to prioritize its therapeutic efficacy because sorafenib was only indicated for differentiated thyroid cancer that was not amenable to curative resection and could not be used for PDTC in our country. Despite the presence of metastatic disease, our patient survived for six years post-diagnosis. Considering that standard treatment doses could not be administered and that the five-year disease-specific survival rate for metastatic PDTC is 34%, this clinical course is considered acceptable [3]. This case underscores the potential survival benefit of lenvatinib in aggressive thyroid cancers, even in the setting of significant renal toxicity. While lenvatinib is a highly effective therapeutic option for PDTC, vigilant monitoring of renal function and proteinuria is crucial. Early identification of renal dysfunction and appropriate intervention, including dose reduction or temporary discontinuation, may help prevent irreversible kidney damage.

## Conclusions

Lenvatinib-induced severe proteinuria in our case was attributed to GMA, as demonstrated by renal biopsy. This underscores the importance of renal monitoring in patients receiving TKI and the possibility of continuing lenvatinib despite the presence of proteinuria. Further accumulation of similar cases and pathological analyses is needed to better understand the renal toxicity spectrum of lenvatinib and to establish appropriate management strategies.
